# Peg Precipitation Coupled with Chromatography is a New and Sufficient Method for the Purification of Botulinum Neurotoxin Type B

**DOI:** 10.1371/journal.pone.0039670

**Published:** 2012-06-28

**Authors:** Yao Zhao, Lin Kang, Shan Gao, Xing Gao, Wenwen Xin, Jinglin Wang

**Affiliations:** State Key Laboratory of Pathogen and Biosecurity, Beijing Institute of Microbiology and Epidemiology, Fengtai District, Beijing, People’s Republic of China; University of Helsinki, Finland

## Abstract

*Clostridium botulinum* neurotoxins are used to treat a variety of neuro-muscular disorders, as well as in cosmetology. The increased demand requires efficient methods for the production and purification of these toxins. In this study, a new purification process was developed for purifying type B neurotoxin. The kinetics of *C.botulinum* strain growth and neurotoxin production were determined for maximum yield of toxin. The neurotoxin was purified by polyethylene glycol (PEG) precipitation and chromatography. Based on design of full factorial experiment, 20% (w/v) PEG-6000, 4°C, pH 5.0 and 0.3 M NaCl were optimal conditions to obtain a high recovery rate of 87% for the type B neurotoxin complex, as indicated by a purification factor of 61.5 fold. Furthermore, residual bacterial cells, impurity proteins and some nucleic acids were removed by PEG precipitation. The following purification of neurotoxin was accomplished by two chromatography techniques using Sephacryl™ S-100 and phenyl HP columns. The neurotoxin was recovered with an overall yield of 21.5% and the purification factor increased to 216.7 fold. In addition, a mouse bioassay determined the purified neurotoxin complex possessed a specific toxicity (LD_50_) of 4.095 ng/kg.

## Introduction

Botulinum neurotoxins (BoNTs), considered as the most toxic substances in nature, are produced by the gram-positive and endospore-forming anaerobic bacteria, *Clostridium botulinum.* These toxins can be divided into seven serotypes (A–G) which are structurally similar, yet antigenically distinct. BoNTs are translated as a single 150-kDa polypeptide chain, which is post-translationally processed by host or ectogenous proteases into a disulfide-linked di-chain toxin consisting of a 50-kDa light chain and a 100-kDa heavy chain protein. Generally, BoNTs are usually associated with non-toxic neurotoxin-associated proteins (NAPs) such as hemagglutinin and non-hemagglutinating proteins that are known as “progenitor” toxins [1]. The composition and sizes of the complexes or progenitor toxins can vary depending on toxin type. Whereas 12 S (300 kD), 16 S (500 kD) and 19 S (900 kD) can be found in type A [2], only 12 S can be detected in type E [3]. Furthermore, BoNT/B complexes can exist in two forms, 12 S and 16 S [1].

Recently, similar to BoNT/A progenitor toxin, BoNT/B has been used for treating patients with strabismus, blepharospasm, nystagmus, facial spasm, spastic aphonia, and many other forms of dystonia [4], [5]. Clinical trials using the type B toxin can alleviate pain and excessive muscle contraction associated with cervical dystonia [6], [7], [8]. Furthermore, BoNT/B exhibited significantly less effects to nearby and relatively distant non-injected muscles compared with BoNT/A when doses were adjusted to produce an equivalent direct effect. These findings suggest that BoNT/B may offer advantages for modulating efficacy and safety of using this neurotoxin, which are concerning issues in cutaneous medicine and surgery.

For type A and B toxins, progenitor toxins are used during treatment because they are easily obtained and are more stable than the neurotoxin itself. Although the treatment is very effective, serious side effects have been observed for some patients using progenitor toxins. It was reported that using neurotoxin alone was better than using progenitor toxin [9]. Generally, the purity of the neurotoxin primarily depends on its intended use in different applications. The highly purified neurotoxin is needed in many applications, such as drug development and the use of the toxin inhibitor as a research tool.

Previously published purification procedures for BoNTs typically includes acid or (NH_4_)_2_SO_4_ precipitations [10], [11] and affinity chromatography [9]. These methods are time-consuming and may be inapplicable for industrial production. Therefore, a new rapid purification process was developed in this study for highly purifying BoNT/B by PEG precipitation and chromatography. The obtained purified BoNT/B can be use for antibody production, a toxoid vaccine, and a licensed drug.

## Results

### Growth Conditions and Toxin Production

**Figure 1:**
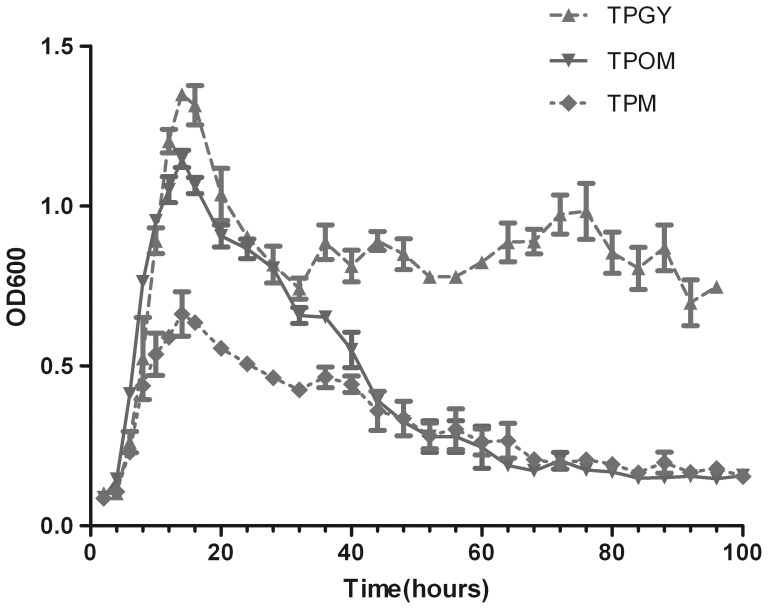
Growth patterns of *C. botulinum* type B strain. It indicates the optical density of the cultures in three media.

The strains both exhibited higher growth rates and reached higher peak cell densities when cultured in TPOM and TPGY (Fig. 1). Cell lysis following stationary phase (12–20 h of growth) occurred in TPOM, TPM and TPGY. However, significant differences in cell lysis were observed when culturing strain in TPGY, TPOM and TPM. Additionally, the strains lysed poorly in TPGY and more extensively in TPM and TPOM.

**Figure 2:**
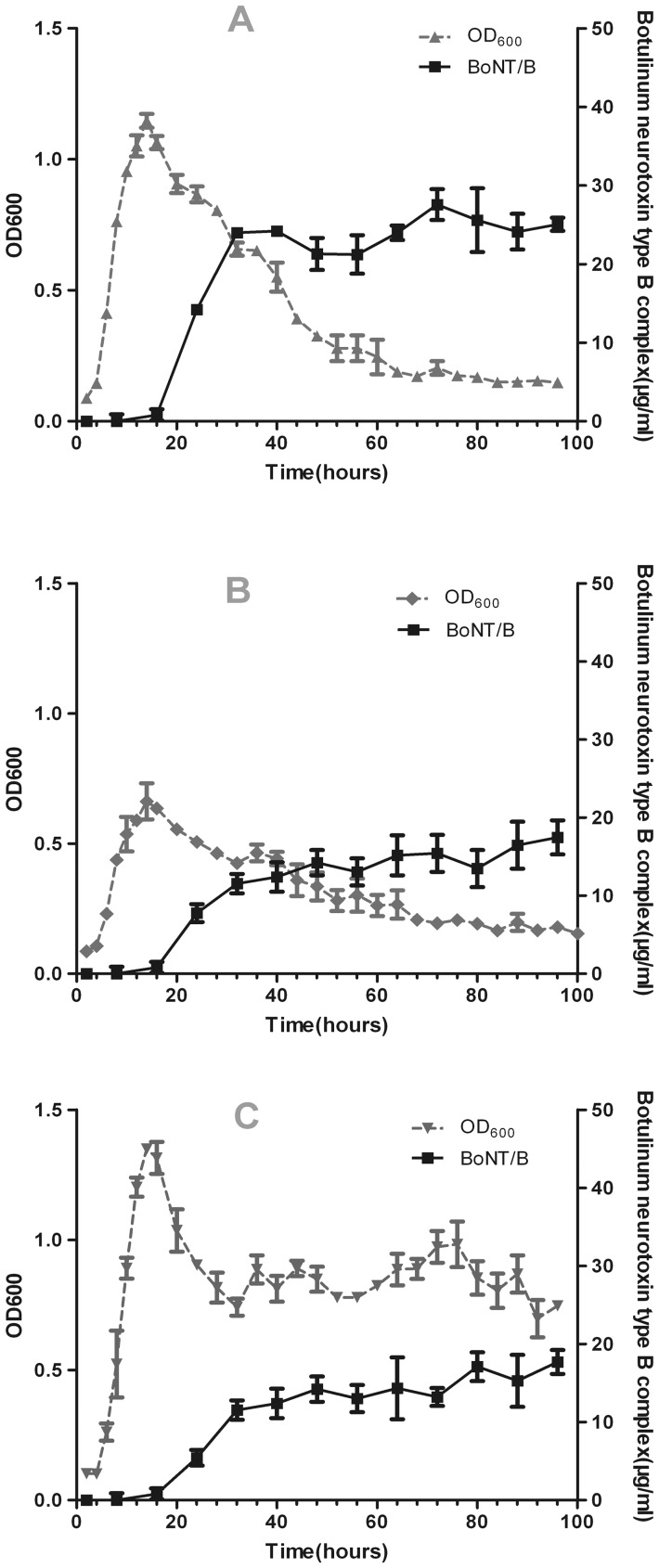
Determination of botulinum toxin type B expression in *C. botulinum* strain grown in three media. A, TPOM; B, TPM; C, TPGY. Botulinum toxin concentration was determined in culture supernatants by ELISA.

The concentrations of the BoNT complex were determined in cell-free culture supernatants at each indicated time point (Fig. 2A–C). For the strain in the three media, maximum extracellular toxin levels were observed after about 32 hours, and extracellular toxin levels continued to increase with more lysis at 20 ∼32 hours. Cell lysis of the strain was much greater in TPOM than in TPM or TPGY at 96 h, and larger quantities of toxin in culture supernatants correlated with extensive cell lysis. Toxin levels by ELISA were approximately 2-fold greater in TPOM than in TPGY or TPM after 96 h of growth (Fig. 2A–C). Overall, the strain in TPOM expressed markedly higher toxin levels than in TPM or TPGY.

### PEG Precipitation

**Figure 3:**
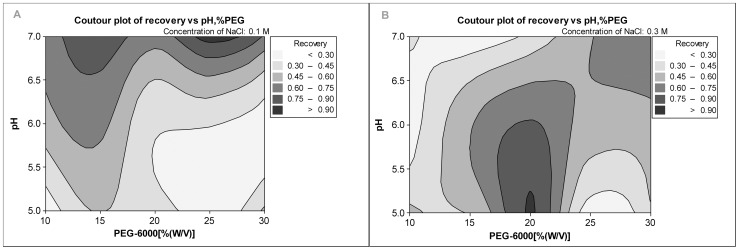
Recovery of BoNT/B by PEG precipitation with various concentrations of PEG at various pH values. A, NaCl concentration: 0.1 M; B, NaCl concentration: 0.3 M. Recovery was defined as the ratio of the amount of BoNT/B measured by ELISA in PEG precipitation to that in the starting material.

After the initial PEG precipitation, residual bacterial cells, nucleic acids and some impurity proteins were removed (data not shown). During the second step of PEG precipitation, highest recovery of BoNT/B occurred using 0.3 M NaCl than 0.1 M NaCl (Fig. 3A–B). For both concentrations of NaCl (0.1 M and 0.3 M), the concentration of PEG used was a primary factor during the recovery and purification of the redissolved BoNT/B within a certain range. However, when 0.1 M NaCl was used during the purification, the pH played a role in the recovery of BoNT/B, which was similarly seen when using 0.3 M NaCl at 20%–30% (w/v) PEG-6000 (Fig. 3B). Therefore, both ionic concentration and pH had influence on the recovery of BoNT/B during PEG precipitation, but the concentration of PEG played a vital role in the recovery. Precipitation with high PEG concentration (25% PEG-6000) resulted in a higher recovery using 0.1 M NaCl and pH 7.0 (Fig. 3A). However, a higher recovery rate occurred at the expense of increased impurities in the redissolved BoNT/B. Therefore, the final optimized condition used 20% (w/v) PEG-6000, pH 5.0 and 0.3 M NaCl. The recovery rate was at 87% and the purification factor was 61.5 fold (Table 1), and parts of the impurity proteins and nucleic acids could be removed using these optimized conditions.

**Table 1:** Purification of the BoNT/B complex

Fraction	Total BoNT/B complex^a^(mg/L)	Toxin titer^b^(MLD/mL)	Yield (%)	Purification^c^ (fold)
Culture supernatant	25	1.5×10^5^	−	1
PEG precipitation	21.75	1.3×10^7^	87	61.5
Size-exclusion chromatography	10.625	2.88×10^6^	42.5	108.5
Hydrophobic interaction chromatography	5.375	1.25×10^7^	21.5	216.7

a The concentration of total BoNT/B complex was determined by a “sandwich” ELISA method using the purified BoNT/B as a standard. And the purified BoNT/B concentration was determined by BCA assay.

b Minimum lethal dose (MLD) was defined as the minimum dose required to kill two mice as described in Materials and Methods.

c The purification factor was estimated by the mouse toxigenicity test of BoNT/B complex.

### Size-exclusion Chromatography (SEC)

**Figure 4:**
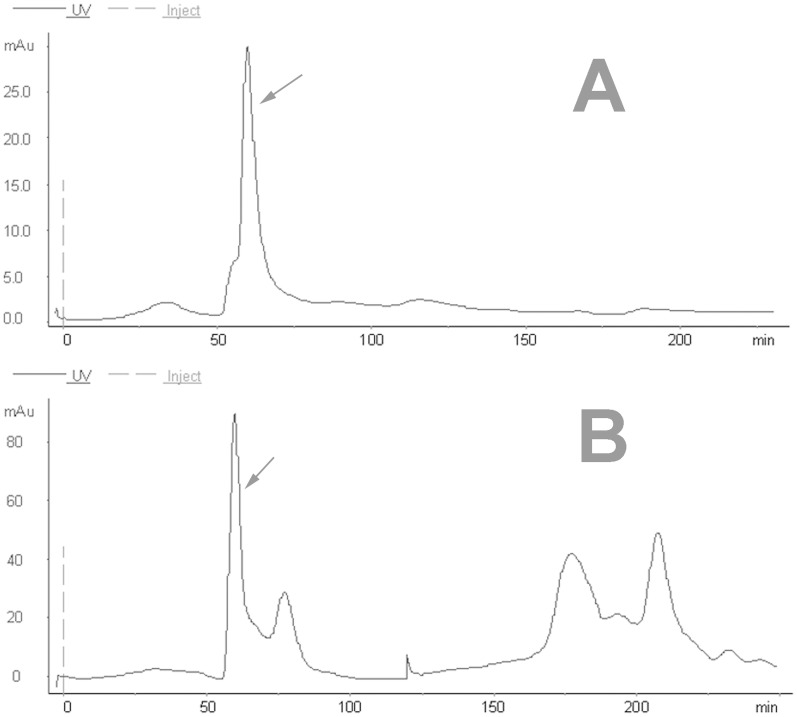
Purification of toxins using size-exclusion chromatography with Sephacryl™ S-100 size column. A, The BoNT/B complex was eluted with buffer at pH 5.8; B, the purification of the BoNT/B was running with buffer at pH 8.0.The arrow shows the activity fraction.

After PEG precipitation, the redissolved protein supernatant was concentrated to enhance resolution and minimize the volume applied to the SEC column. The data showed that the SEC elution profile included a single–larged and well-formed early eluting peak corresponding to the high molecular weight BoNT/B complex at pH 5.8 (Fig. 4A). However, at pH 8.0, the SEC elution profile included a high early eluting peak corresponding to the high molecular weight BoNT/B, and some small peaks corresponding to contaminants (Fig. 4B). The fraction contained a toxicity of 2.88×10^6 ^MLD/mL. The yield of SEC was up to 42.5%, and the purification factor was 108.5 fold (Table 1). The remaining impurities were removed by employing hydrophobic interaction chromatography (HIC).

### Hydrophobic Interaction Chromatography (HIC)

**Figure 5:**
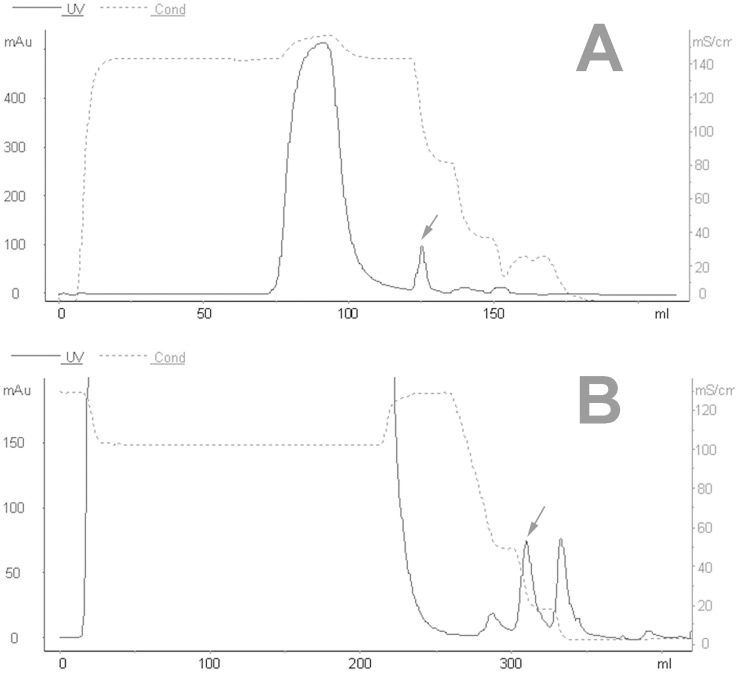
Further purification of toxins using hydrophobic interaction chromatography. Pooled fractions obtained from the Sephacryl™ S-100 size column (Fig. 4) were applied to a phenyl HP column. A, The BoNT/B complex was eluted at pH 5.8; B, the BoNT/B was purified at pH 8.0. The arrow shows the activity fraction.

**Figure 6:**
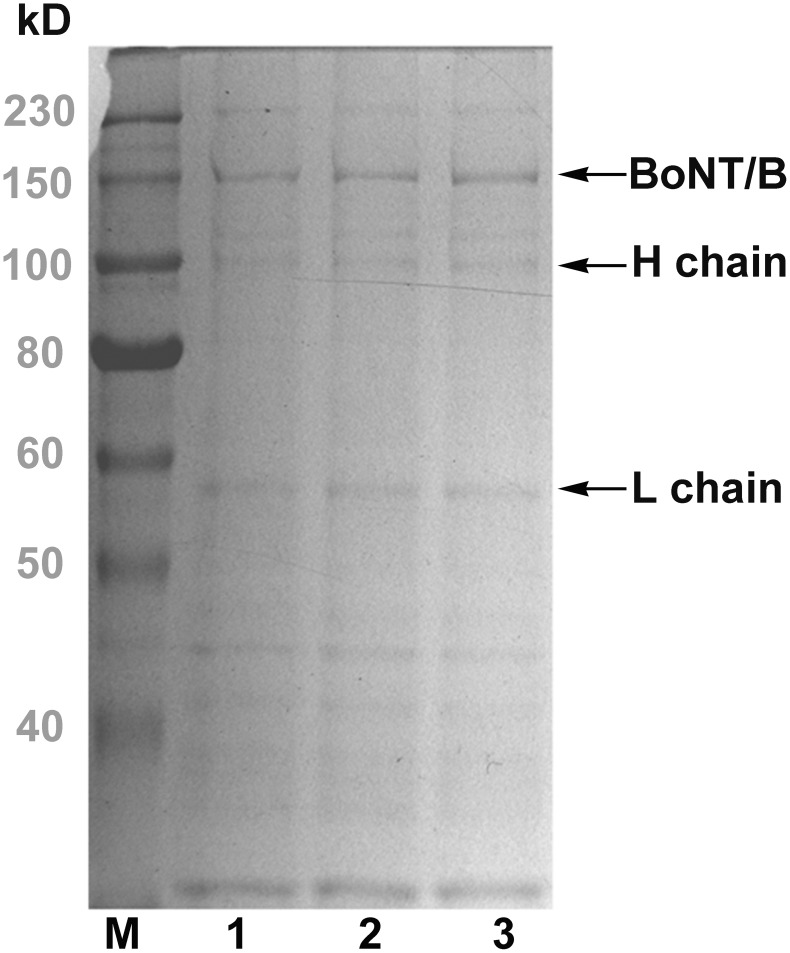
SDS–PAGE analysis of the purified BoNT/B complex. 1∼3, BoNT/B complex (diluted 1:2 with sample buffer containing DTT) after purification by hydrophobic interaction chromatography column; M, high molecular markers.

The fractions containing the interested protein were pooled and passed through hydrophobic interaction chromatography using a Phenyl HP column. An optimal pH of 5.8 was used to purify the BoNT/B complex. The column was washed with 4∼5 bed volumes of buffer A to remove all the unbound proteins. The results showed that an optimized stepwise gradient of 0.6 M, 0.2 M, and 0 M of ammonium sulphate into a Phenyl HP column (Fig. 5A) was most effective for isolating the BoNT/B complex. The fraction containing the BoNT/B complex was eluted using 0.6 M of ammonium sulphate. The fractions obtained were analyzed by SDS-PAGE (Fig. 6).

**Figure 7:**
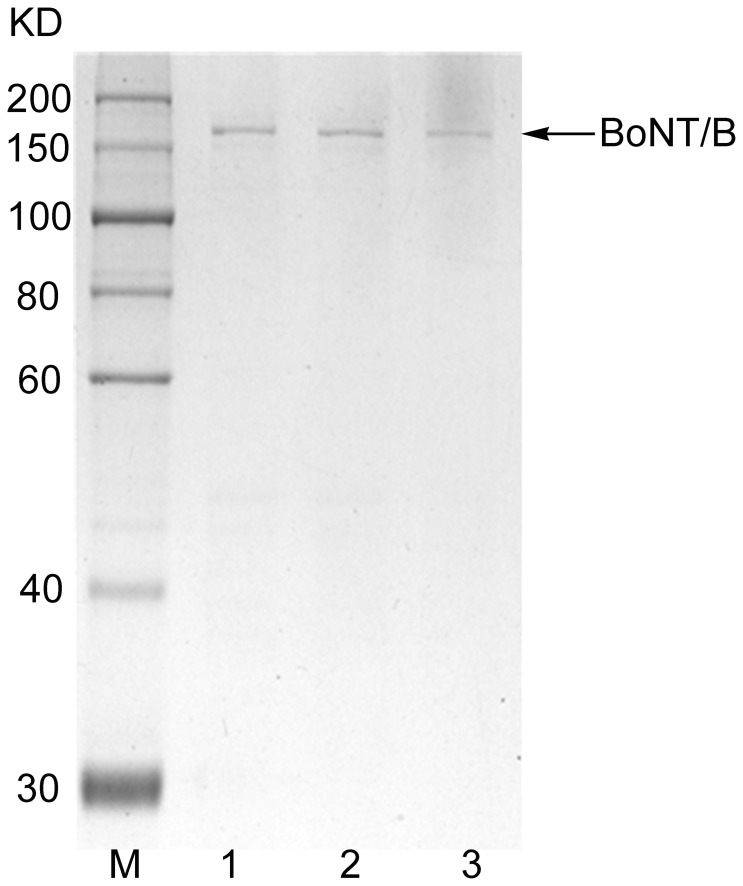
SDS–PAGE analysis of the purified BoNT/B. 1∼3, BoNT/B after purification by hydrophobic interaction chromatography column; M, markers.

An optimal pH of 8.0 was used to purify BoNT/B. The retention and elution of BoNT/B was optimized with a further increase in its purity by decreasing the levels of ammonium sulphate using a gradient from 1 M to 0 M. The corresponding chromatograms are given in Fig. 5B. The BoNT/B was eluted at an ammonium sulfate concentration between 0.8 and 0.6 M. Most of impurities were eluted in the flow-through fractions and at the first major peak. The purity was determined at 95% by SDS-PAGE (Fig. 7).

In this study, HIC was used as the final purification step. The final product contained a toxicity of 1.25×10^7 ^MLD/mL. The final recovery of the purification was up to 21.5% and the purification factor was 216.7 fold (Table 1).

### Mouse Toxicity of BoNT/B Complex

**Figure 8:**
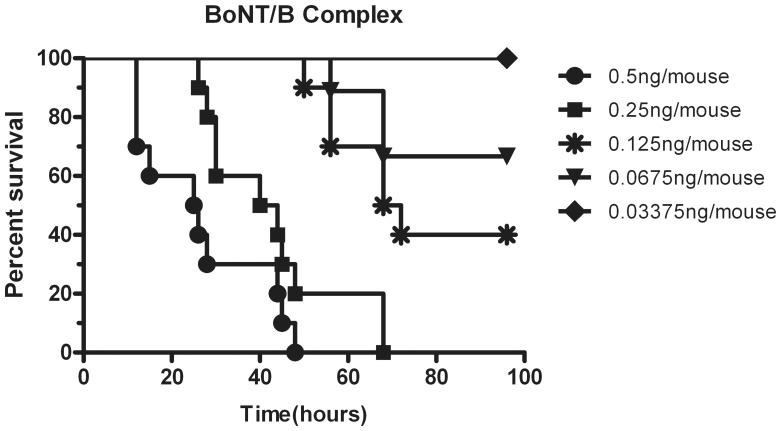
Survival curves of mice treated i.p. with BoNT/B complex. Percent survival was plotted over time. Groups of ten mice were used at each dosage level and five dose levels were tested per experiment as described in Materials and Methods. The animals were observed for death over a period of 4 days.

The LD_50_ of BoNT/B using Karber’s method was 4.095 ng/kg (about 0.0901 ng/mouse) with a 95% confidence limit between 3.077 and 5.468 ng/kg. Deaths occurred in most cases between 12 and 48 h after dosing (Fig. 8), with mortalities also observed at a low dose of 0.125 and 0.0675 ng/mouse after 48 h. At a high dosage (0.5 ng/mouse, filled circles), BoNT/B caused more rapid lethality compared to the low dosage. Surviving mice that received a dosage of BoNT/B displayed classic symptoms of botulism including ruffled fur, wasp-waist, hind limb paralysis and labored breathing compared to control mice [12], [13]. These results provided information for the toxicity of BoNT/B given by intraperitoneal injection.

## Discussion

It is necessary that BoNT/B can be consistently highly produced for its purification. The growth and the production of biologically active BoNTs and BoNT complexes are known to vary with the strains of *C. botulinum*, medium compositions, and culture conditions [14]. Inconsistent batch-to-batch production of the same strain has been described, including *C. botulinum* type A [15]. To better optimize the conditions for BoNT/B production, the kinetics of *C. botulinum* strain growth and BoNT/B production is needed to be clearly defined. In this study, the growth of *C. botulinum* strain and BoNT/B production differed in TPOM, TPGY and TPM. A higher concentration of BoNT/B was obtained from TPOM supernatant (see Fig. 2), which correlated with a higher rate of cell lysis.

PEG precipitation is a crude and non-specific technique that separates proteins by their solubility. PEG acts as an inert solvent sponge and reduces solvent availability. Protein concentration is increased upon increasing concentrations of PEG until the solubility is exceeded and precipitation occurs [16]. In this study, the crude BoNT/B was extracted by PEG precipitation. PEG precipitation not only removes the contaminant, but is stable as PEG precipitates that can be resuspended to concentrate the dissolve BoNT/B. To achieve this, we used 20% (w/v) PEG-6000, pH 5.0 and 0.3 M NaCl, and reported a recovery rate of BoNT/B complexes up to 87%.

Notably, BoNT/B is produced as BoNT/B complexes in which the neurotoxin component is non-covalently linked with non-toxic proteins. As mentioned previously, BoNT complexes are stable only at slightly acidic pH range of 5∼7 [17], [18], and can dissociate in alkaline solutions (pH values >7) [19], [20]. In this study, for the purification of BoNT/B and BoNT/B complexes, the first purification step was a SEC at pH 8.0 and pH 5.8, respectively. At pH 8.0, the complex dissociated and could partly be separated within this step (Fig. 4B). Indeed, in this step, some small impurity proteins and parts of nucleic acid were removed with a Sephacryl™ S-100 size column.

Furthermore, for further purification to remove most of the nucleic acids and some impure proteins, HIC was performed with a phenyl HP column. In HIC, both the hydrophobic ligand types and salt concentration in solution could influence chromatographic resolution. HIC has also been used as an effective purification step for high-value biomolecules such as monoclonal antibodies [21], [22]. In contrast to other chromatographic techniques, HIC is viewed as a relatively gentler procedure, leading to its widespread use in downstream purification of biopharmaceuticals [23]. Optimal purification of BoNT/B and BoNT/B complexes was observed using a stepwise and gradient elution by a Phenyl HP column. The purity was determined to be 95% by SDS-PAGE (Fig. 7).

A new rapid purification process was developed in this study for highly purified BoNT/B by PEG precipitation and chromatography that required less time (9 days) than a previously published method (at least 11 days) [11]. Compared with acid or (NH_4_)_2_SO_4_ precipitation, PEG precipitation is a gentle method to extract proteins and contribute for protecting protein activity. Particularly, PEG precipitation can remove residual bacterial cells, and parts of impurified proteins and nucleic acids. This new efficient procedure could be applicable for the purification of other types of toxin.

The “gold standard” for the detection and measurement of BoNTs is the intraperitoneal mouse bioassay [13], [24]. In this study, this bioassay was used to test the toxicity of purified the BoNT/B complex. The data indicated that a higher dose killed more effectively and sufficiently than a lower dose, and the LD_50_ of BoNT/B calculated by the Karber’s method was 4.095 ng/kg. *These results provided information for the toxicity of BoNT/B given by intraperitoneal injection, but it had been reported that the toxicity of BoNT/B is lower than that of BoNT/A* [*25*], *and we obtained a similar result with the purified BoNT/B.*

In summary, the kinetics of *C. botulinum* strain growth and BoNT/B production were optimized for maximum yield, and TPOM was the more suitable culture for its growth and production. The purification method for BoNT/B and BoNT/B complex was standardized after PEG precipitation and two chromatographic runs under various conditions. PEG precipitation coupled with chromatography is a rapid and efficient method to purify BoNT/B and its associated complex compared to acid or (NH_4_)_2_SO_4_ precipitation. Therefore, it is hopeful that this method could be used for the purification of other types of toxin, vaccine production and synthesis of BoNT for pharmaceutical purposes, as well as for the fundamental studies of the structure and function of BoNT.

## Materials and Methods

### Chemicals and Bacterial Strain

Polyethylene glycol (PEG, MW: 6000 kDa) was purchased from Merck. Ammonium sulfate ((NH_4_)_2_SO_4_), potassium phosphate dibasic anhydrous (K_2_HPO_4_), sodium phosphate monobasic anhydrous (NaH_2_PO_4_) and sodium chloride (NaCl) were obtained from Sigma. All other chemicals were of analytical grade.

The type B botulism antitoxin (equine) was provided by the Lanzhou Institute of Biological Products, Lanzhou, China. The *C. botulinum* strain that could produce BoNT/B was provided by the State Key Laboratory of Pathogens and Biosecurity, Beijing, China and used in this study.

### Production of the BoNT/B

The *C. botulinum* type B strain was cultured in three different types of media, including TPYG (5% trypticase peptone, 0.5% bacto peptone, 0.4% glucose, 2% yeast extract and 0.1% L-cysteine, pH 7.4), toxin production medium (TPM, 2% casein hydrolysate, 1% yeast extract, and 0.5% glucose, pH 7.2), and optimized medium of toxin production (TPOM, yeast extract 1%, casein hydrolysate 2%, Na-thioglycolic acid 0.05%, L-cysteine HCL 0.1% and glucose 0.5% pH 7.2).

The *C. botulinum* type B strain stored at −80°C was inoculums (0.5 ml) for 25 ml of cooked meat media (peptone 3%, yeast extract 0.5%, beef extract 0.5%, glucose 0.3%, NaH_2_PO_4_ 0.5%, pH 7.2∼7.4) incubated at 37°C. Three 1 L glass carboys each containing 1 L of three different culture mediums were autoclaved 20 min. The three carboys cooled down overnight to 30°C were individually inoculated with 10 ml of active culture growing in the cooked meat medium and incubated undisturbed for 96 hours at 30°C.

The cultures were sampled every 2 hours for 28 hours and every 4 hours in subsequent 68 hours. At each time point, the OD_600_ was measured and samples were analyzed for detection of BoNT/B by Enzyme-linked immunosorbent assay (ELISA).

### Extraction of the BoNT/B using PEG Precipitation

After 96 hours of culture, BoNT/B was extracted from cultured media using the following steps. First, the condition of initial PEG precipitation was 5% (w/v) PEG-6000 and pH 6.8. After 5∼6 hours of precipitation, the culture media was centrifuged at 10,000 g for 20 min, and pellets were decanted. Second, for the supernatant from the initial PEG precipitation, the effects of PEG concentration, ionic intensity and PH on PEG precipitation were optimized by full factorial experiment. The pH of the supernatant was adjusted to the desired level with 1 mol/L Tris-HCl using a pH electrode (Mettler Toledo SG2). The PEG 6000 was added very slowly with stirring at a 50% (w/v) stock solution to the desired final concentration. After 30 min of stirring, the supernatant was stored overnight at 4°C. The supernatant was centrifuged at 10,000 g for 20 min. The supernatant was decanted and the protein pellets were collected. Pellets were resuspended in one-third of the initial volume in a solution of 50 mM Tris–Bis (pH 5.8 for production of BoNT/B complex and pH 8.0 for BoNT/B). Undissolved protein was then removed by a second centrifugation at 10,000 g for 15 min. These conditions must be carefully followed for reproducible results.

### Size-exclusion Chromatography (SEC)

If otherwise stated, ÄKTA FPLC and resins for chromatographic purification were supplied by GE Healthcare. All chromatography steps were performed at room temperature.

After PEG precipitation, the concentrated and pre-purified and resuspended toxin supernatant obtained in the precipitation step was subjected to size-exclusion chromatography using a HiPrep Sephacryl™ S-100 column. The running buffer for this column was 50 mM Tris–Bis, 150 mM NaCl, and the pH of the buffer was 5.8 and 8.0 for the purification of BoNT/B and BoNT/B complex, respectively. Fractions were collected from the column at 1 ml/min. Their absorbance was measured at 280 nm, and the sample was analyzed by SDS-PAGE and ELISA, as described below.

### Hydrophobic Interaction Chromatography (HIC)

After SEC, fractions containing the toxin were subjected to hydrophobic interaction chromatography (HIC) using a HiTrap™ Phenyl HP 5- ml column that equilibrated with 3∼4 bed volumes of buffer A (50 mM Tris–Bis, 0.05 mM EDTA, pH 5.8 or 8.0, and 1 M ammonium sulfate). Flow rate was 5 ml/min. In order to purify the BoNT/B and BoNT/B complex, the pH of the buffer and elution condition were optimized. After washing the column with starting buffer A, the bound protein was eluted either by multi-step elution (e.g. 700, 600, 500, 200 and 0 mM ammonium sulfate) or by gradient elution using a 1∼0 M gradient of ammonium sulfate in 50 mM Tris–Bis buffer containing 0.05 mM EDTA. The fractions obtained were examined at 280 nm, and the sample was analyzed by SDS-PAGE and ELISA.

### Purified Complex Toxin and Neurotoxin Analysis by SDS-PAGE

The purity of botulinum toxins in purified or crude toxin samples was estimated by SDS-PAGE. A 2% stacking and 10% separating vertical gel was used in the Mini Protean II-system (Bio-Rad) with high molecular weight marker (NEB) as a standard. The separation was run at a constant voltage of 180 V for approximately 50 min. Gels were Coomassie-stained and digital images were analyzed using BandScan software.

### Detection and Quantification of BoNT/B using ELISA

To calculate the overall recovery of the toxin, the toxin concentration was determined by a “sandwich” ELISA method. The BoNT/B is captured on the plate by the botulinum antitoxin. A polyclonal antibody specific for BoNT/B from our laboratory [26] is then added into the plate, and can be detected by HRP-conjugated goat anti-mouse IgG. Finally, the plate was developed by adding a substrate to produce the colorimetric reaction, which read the absorbance at 450 nm using the SpectraMAX Microplate Reader (Molecular Devices). The absorbance indicates the quantity of BoNT/B in the sample using the purified BoNT/B as a standard. The determination of purified BoNT/B and total protein concentration use BCA assay. BCA Protein Assay Kit (Pierce) was used with bovine serum albumin as the protein standard.

### Mouse Bioassay

The biological activity of the BoNT/B complex solutions was determined in BALB/c mice weighing 18∼22 g, that were randomized and inoculated intraperitoneally (i.p.) with 500 µl of sample. Groups of ten mice were used at each dosage level and four or five dose levels were tested per experiment. For nicking of the neurotoxin, trypsin was added to a final concentration of 20 µg/ml mixed and incubated at 37°C for 30 min. Serial 2-fold dilutions were then made in gelatine-phosphate-buffer (GPB: 50 mM PBS, 0.2% gelatin, and pH 6.8). The animals were observed for death over a period of 4 days. 50% lethal dose (LD_50_) values were calculated using the Karber’s method [27]. Minimum lethal dose (MLD) was defined as the minimum dose required to kill two mice [13]. A survival curve of mice treated i.p. with BoNT/B complex was determined using the Prism 4 statistics software (Graph Pad Software Inc.).

All mice were housed in pathogen free conditions with free access to food and water. The animal use protocol was conducted in compliance with the Guide for the Care and Use of Laboratory Animals and the Association for Assessment and Accreditation of Laboratory Animal Care International, and including the relevant local animal welfare bodies in China. In addition, the permit number of all animal work was SCXK-(JUN) 2007-004 that approved by the animal ethics committee of Beijing Institute of Microbiology and Epidemiology.
