# Correction: PEG Precipitation Coupled with Chromatography is a New and Sufficient Method for the Purification of Botulinum Neurotoxin Type B

**DOI:** 10.1371/annotation/d00094ef-aac0-43ce-bba6-50d5a1cdbb37

**Published:** 2012-09-20

**Authors:** Yao Zhao, Lin Kang, Shan Gao, Xing Gao, Wenwen Xin, Jinglin Wang

There was a formatting error in the title. The correct title is: PEG Precipitation Coupled with Chromatography is a New and Sufficient Method for the Purification of Botulinum Neurotoxin Type B. 

The correct citation is: Zhao Y, Kang L, Gao S, Gao X, Xin W, et al. (2012) PEG Precipitation Coupled with Chromatography is a New and Sufficient Method for the Purification of Botulinum Neurotoxin Type B. PLoS ONE 7(6): e39670. doi:10.1371/journal.pone.0039670 

